# Evaluating Genomic Testing in Pediatric Inborn Errors of Immunity: An Optimal Model of Care

**DOI:** 10.1007/s10875-026-02057-3

**Published:** 2026-08-06

**Authors:** Emily DeBortoli, Tenielle Clinch, Larissa Vaz-Gonçalves, Lucy Burbury, Cian Thomas, Meg Jeppesen, Alberto Pinzon-Charry, Mariana Melo, Anna Sullivan, Matthew F. Hunter, Jane Peake, Aideen McInerney-Leo, Peter McNaughton, Tatiane Yanes

**Affiliations:** 1 Frazer Institute, Dermatology Research Centre, University of Queensland, Brisbane, Queensland Australia; 2 Queensland Paediatric Immunology and Allergy Service, Children’s Health Queensland, Brisbane, Queensland Australia; 3 Clinical Support Queensland, Pathology Queensland, Brisbane, Queensland Australia; 4 Faculty of Health, Medicine and Behavioural Sciences, University of Queensland, Brisbane, Queensland Australia; 5 School of Environment and Science, Griffith University, Brisbane, Queensland Australia; 6 Department of Metabolic Medicine, Children’s Health Queensland, Brisbane, Queensland Australia; 7 Department of Paediatrics, Monash University, Melbourne, Victoria Australia; 8 Department of Paediatrics and Child Health, University of Queensland, Brisbane, Queensland Australia

**Keywords:** Inborn errors of immunity, Primary immunodeficiencies, Genomic, Model of care, Pediatrics, Immunology

## Abstract

**Purpose:**

While genomic testing is integral to the management of pediatric inborn errors of immunity (IEI), few studies have examined strategies to support its optimal integration into routine healthcare. Thus, this study aimed to: (i) describe a pediatric IEI cohort and genomic testing practices, and (ii) assess the impact of implementing a multidisciplinary genomic model of care (MoC) on service outcomes.

**Methods:**

A comprehensive chart audit was conducted for patients (≤ 18years) who received IEI genomic testing in Queensland, Australia, from 2017 to 2025. Descriptive analyses captured demographics, clinical characteristics, genomic testing and results, and management outcomes. Inferential analyses assessed changes in genomic practices pre-MoC (< 2021) and post-MoC (≥ 2021).

**Results:**

322 patients met eligibility criteria (*n* = 481 genomic tests). Diagnostic yield (mean:26.7%) varied by testing indication, with the highest rate among phagocytic defects (*n* = 4/4;100%) and severe combined immunodeficiency (*n* = 8/10;80.0%). Very early-onset inflammatory bowel disease had the lowest diagnostic yield (*n* = 3/68;4.4%), prompting local changes to testing criteria. Among patients with an IEI molecular diagnosis (*n* = 79), 89.9% received a management change recommendation. Genomic testing was widely used pre-MoC (*n* = 259 genomic tests). All outcomes significantly improved from pre-MoC to post-MoC (*p* < 0.05, chi-square test): duplicate testing eliminated (13.5% to 0.0%); variants of uncertain significance reduced (37.7% to 7.1%); informed consent documentation improved (70.5% to 88.4%); and diagnostic yield increased (14.5% to 27.4%).

**Conclusion:**

Implementing a multidisciplinary genomic MoC in pediatric IEI improved clinical and health service outcomes. Building on these findings, this study offers practical recommendations for integrating genomic testing into routine pediatric IEI practice, to enhance diagnostic pathways and health service performance.

**Supplementary Information:**

The online version contains supplementary material available at https://doi.org/10.1007/s10875-026-02057-3.

## Introduction

Inborn errors of immunity (IEI) comprise a heterogeneous group of genetic conditions that affect the immune system, causing increased susceptibility to infections, autoimmunity, autoinflammation, malignancies, and/or allergies [1]. These conditions most commonly present in the pediatric setting, where they are often associated with serious and life-limiting complications [2]. While IEI are individually rare, the collective prevalence of such conditions is estimated to be between 1:1000 and 1:5000, posing a significant disease burden [3]. IEI are classified according to the primary immunological mechanism that is disrupted or dysregulated, with the International Union of Immunological Societies (IUIS) most recent report recognizing ten categories [4].

Genomic testing has become an essential aspect of IEI care, with more than 500 associated genes characterized to date [1]. Establishing a molecular diagnosis can enable targeted therapeutic interventions and initiation of curative treatment. IEI treatment modalities include hematopoietic stem cell transplantation (HSCT), immunoglobulin replacement therapy (IRT), gene therapy, and prophylaxis therapies (e.g., antimicrobials and immunomodulators) [5]. Curative therapies are most effective when delivered early in the disease course prior to the onset of infection and/or organ damage, thus increasing the importance of timely molecular diagnosis [5]. A molecular diagnosis can also inform reproductive decisions, facilitate identification of at-risk relatives, mitigate psychological distress among families, and reduce related healthcare expenditure [5, 6].

Although the clinical and personal utility of IEI genomic testing is widely recognized, progress in developing models of care to support the optimal integration of testing into routine immunology healthcare remains limited [7, 8]. There exists a lack of guidelines to support patient identification, consent processes, test selection, result communication, and familial considerations [9, 10]. Similarly, few studies have examined the ethical, legal, and social implications of genomics in IEI, and how these factors influence testing practices [8, 11]. The absence of a dedicated genomic MoC can contribute to inefficiencies in healthcare, such as through missed opportunities for diagnostic testing, delays in the timely delivery of targeted therapies, and limited consideration of at-risk relatives [12].

To address such challenges, a multidisciplinary genomic MoC was developed and implemented for pediatric IEI at the Queensland Pediatric Immunology and Allergy Service (QPIAS), Queensland Children’s Hospital (QCH); an Australian tertiary pediatric hospital [13, 14]. The QPIAS provides publicly funded pediatric immunology healthcare to the Queensland population, constituting an expansive geographical area (> 1.7 M km^2^), with a population of around 5.1 million [15]. Key features of this MoC include: (i) monthly state-wide multidisciplinary team (MDT) meetings, attended by pediatric and adult immunologists, genetic counselors, clinical geneticists, genetic and immunology pathologists, nursing staff, and other specialist doctors, to review genomic testing practices, result interpretation, and clinical and familial implications of molecular findings; [13] (ii) a genetic counsellor embedded in the service; (iii) dedicated role and funding for a clinical immunologist to specialize in genomics; (iv) trio-whole exome sequencing (WES) via virtual panel analysis as a front-line diagnostic test; [16] (v) collaborative engagement with the statewide pathology service (i.e. Pathology Queensland); and (vi) strong research collaborations with the Clinical Immunogenomics Research Consortium Australasia (CIRCA) [17].

This study is part of the BRIDGE (**B**uilding **R**esources for **I**mmunology **D**iagnostic **GE**nomics) project, which aims to optimize pediatric IEI genomic testing practices by considering the needs of children, families, healthcare providers, and health services. Internationally, several IEI registries have aided in the categorization of genomic data, therapeutic interventions, and clinical outcomes [18]. However, these registries lack data regarding service delivery, limiting the capacity to inform genomic testing practices. Furthermore, there is no Australian data describing local pediatric IEI cohorts.

Thus, in the context of a real-world clinical setting, this study aimed to: (i) characterize an Australian IEI pediatric cohort and genomic testing practices, and (ii) assess the impact of implementing a genomic MoC in this setting and identify areas for service improvement. Ultimately, such findings will inform future clinical guidelines and genomic testing practices, thereby leading to an enhancement in clinical care of pediatric IEI patients.

## Materials and Methods

### Ethics Approval

This study received ethics approval from QCH (HREC/24/CHQ/106207) and the University of Queensland (2024/HE000776) and Queensland Public Health Act approval (PHA/106207).

### Patient Eligibility and Data Collection

Individuals were eligible for inclusion if they had IEI genomic testing from 2017 to 2025 and were aged ≤ 18 years at the time of testing. There was no exclusion criteria based on the modality of genomic testing or testing outcome (i.e., positive, negative, variant of uncertain significance (VUS)). Patients were excluded if the genomic test report could not be located, and therefore, results could not be confirmed. Eligible patients were identified via three pathways: (i) genomic testing ordered by a QPIAS clinician (list generated by Pathology Queensland), (ii) private laboratory online accounts for QPIAS clinicians, and (iii) review of internal QPIAS files to identify additional avenues of testing and to capture patients who had been transferred to another service for clinical care post-genomic testing.

A genomic testing chart audit was completed for all eligible patients by authors T.C., A.S., P.M., and T.Y., with the following data collected: demographic information, instances of genomic testing, genomic testing details, genomic testing result, and outcomes of genomic testing (Supplementary Table 1). Instances of genomic testing ordered by both immunology and non-immunology specialists were included to reflect real-world practices. Due to the lack of accurate documentation of research participation, identifying patients who underwent research testing required collaboration with the clinicians involved to confirm testing and outcomes. All genomic test data was documented in a RedCap database hosted at QCH. Additionally, author L.B. reviewed 10% of RedCap entries to ensure data accuracy and assist with complex cases. Any discrepancies between authors were resolved through discussion until consensus was achieved.

### Data Categorization and Analysis

Basic descriptive statistics summarize demographic information, indications for testing, number and/or timing of genomic tests, ordering specialty, test modality, results, and patient management recommendations. Geographical remoteness was assessed using the Australian Statistical Geography Standard (ASGS), [19] and socio-economic status was determined using the Socio-Economic Indexes for Areas (SEIFA),[20] both calculated via postcode data according to the Australian Bureau of Statistics. The cohort distribution based on the ASGS and SEIFA was compared to the 2021 Queensland census data using chi-square analysis [15].

Indications for testing were categorized based on the Australasian Society for Allergy and Immunology (ASCIA) guidelines [21, 22]. Testing modality categories included, chromosomal microarray (CMA), single gene, panel, virtual panel, WES, whole genome sequencing (WGS), familial testing, research testing, and other genomic tests (e.g., mitochondrial DNA (mtDNA) point mutation analysis, fragile X testing, somatic analysis, karyotype). Virtual panels involve filtering WES or WGS data against a curated gene list, which allows for reflex analysis to expanded testing as required. Comparatively, traditional gene panels involve targeted sequencing of a fixed list of genes [23]. For patients who received > 1 panel or virtual panel test, subsequent tests were categorized as a new panel, an expanded panel (i.e., inclusion of additional genes based on updated knowledge), or panel re-analysis (i.e., reviewal of previously generated sequencing data). All panel and virtual panel tests were categorized according to the Australian PanelApp panel that most closely aligned with the genes tested. PanelApp Australia is an open-access platform that hosts curated gene panels for specific phenotypes [16, 24]. PanelApp includes several panels tailored to specific IEI presentations (e.g., autoinflammatory disorders), as well as an ‘immunological disorders super panel’, which combines all IEI related panels. QPIAS clinicians actively contribute to the development and refinement of these panels, which are currently applied to virtual panel tests within the MoC.

In line with American College of Medical Genetics and Genomics (ACMG) guidelines, [25] diagnostic results were limited to pathogenic and likely pathogenic variants confirmed in an accredited clinical genetics laboratory (e.g., National Association of Testing Authorities (NATA) in Australia) [26]. Diagnostic results were assessed to investigate the impact on patient management and were classified according to the IUIS categories [4]. Major management changes included recommendations for HSCT, IRT, other targeted treatment and management, prophylaxis monitoring, or changes to HSCT conditioning regimen. Minor management recommendations pertained to changes to existing monitoring. Patients who underwent multiple genomic tests were reviewed to describe the testing pathway and identify potential redundancies (e.g. duplicate testing).

The impact of the MoC on genomic testing practices was compared between pre-MoC (< 2021) and post-MoC (≥ 2021) using chi-square analysis. Statistical significance was inferred at *p* < 0.05. To further assess genomic testing practices, the pre-MoC and post-MoC periods were grouped into four stages, aligned with changes in genomic testing access and implementation of the MoC:

i) ≤ 2018 pre-MoC with limited access to genomic testing.
ii) 2019-20 pre-MoC with introduction of point-of-care genomic testing.
iii) 2021-22 post-MoC encompassing implementation and pilot testing of the MoC.
iv) 2023-25 post-MoC including formal establishment of the MoC.

## Results

### Cohort Demographics

Of the 362 patients identified, 40 were excluded as they did not meet study eligibility criteria (*n* = 32 genomic test could not be verified, *n* = 1 genomic testing initiated > 18 years, and *n* = 7 received only non-IEI genomic testing). Thus, 322 patients were included in the study. While all patients were alive at the time of genomic testing, 10 (3.1%) were deceased at the time of data collection (Table 1). The mean age at first genomic testing was 5.9 years (range: 0–17 years), and 173 (53.7%) of the cohort were male, based on biological sex. Most patients resided in a major city (*n* = 233;72.4%) or inner regional area (*n* = 38;11.8%) as per ASGS. Almost half of the cohort resided in an area classed by SEIFA as socio-economically advantaged: most advantaged (*n* = 57;17.9%) or advantaged (*n* = 90;28.3%). Patient distribution was comparable to the general Queensland population (*p* > 0.05), and 17 patients resided in Northern New South Wales. The most frequent indications for genomic testing were very early onset inflammatory bowel disease (VEO-IBD) (*n* = 68;21.1%), immune dysregulation (*n* = 36;11.2%), atypical infection (*n* = 32;9.9%), predominantly antibody deficiency (*n* = 32;9.9%), and autoinflammation (*n* = 28;8.7%). The indication for testing could not be determined for 45 patients (14.0%). Notably, two patients were first identified via a positive severe combined immunodeficiency (SCID) newborn screening test (implemented May 2023).

**Table 1:** Cohort demographic

Characteristic		*n* (%)	*n* (%)
Biological sex			
	Female	149 (46.3)	
	Male	173 (53.7)	
Age at first genomic test (years old)			
		Mean:5.9 (range: 0–17)	
Living status			
	Alive	312 (96.9)	
	Deceased	10 (3.1)	
Geographical classification (ASGC)		*n* (%)	Qld population^a^
	Major city	233 (72.4)	3,426,582 (67.0)
	Inner regional	38 (11.8)	920,816 (18.0)
	Outer regional	29 (9.0)	684,865 (13.4)
	Remote	1 (0.3)	33,465 (0.7)
	Very remote	4 (1.2)	46,338 (0.9)
	Outside of Queensland	17 (5.3)	NA
Socio-economic status (SEIFA)			
	Most advantaged	57 (17.9)	852,726 (16.7)
	Advantaged	90 (28.3)	1,276,043 (24.9)
	Middle	74 (23.3)	1,223,084 (23.9)
	Disadvantaged	42 (13.2)	796,027 (15.6)
	Most disadvantaged	42 (13.2)	964,186 (18.9)
	Outside of Australia	17 (5.3)	NA

^a^Cohort is proportional to the Queensland population (*p* > 0.05, chi-square test). Combined remote and very remote as each *p* < 0.5

### Genomic Testing Practices

**Fig. 1:**
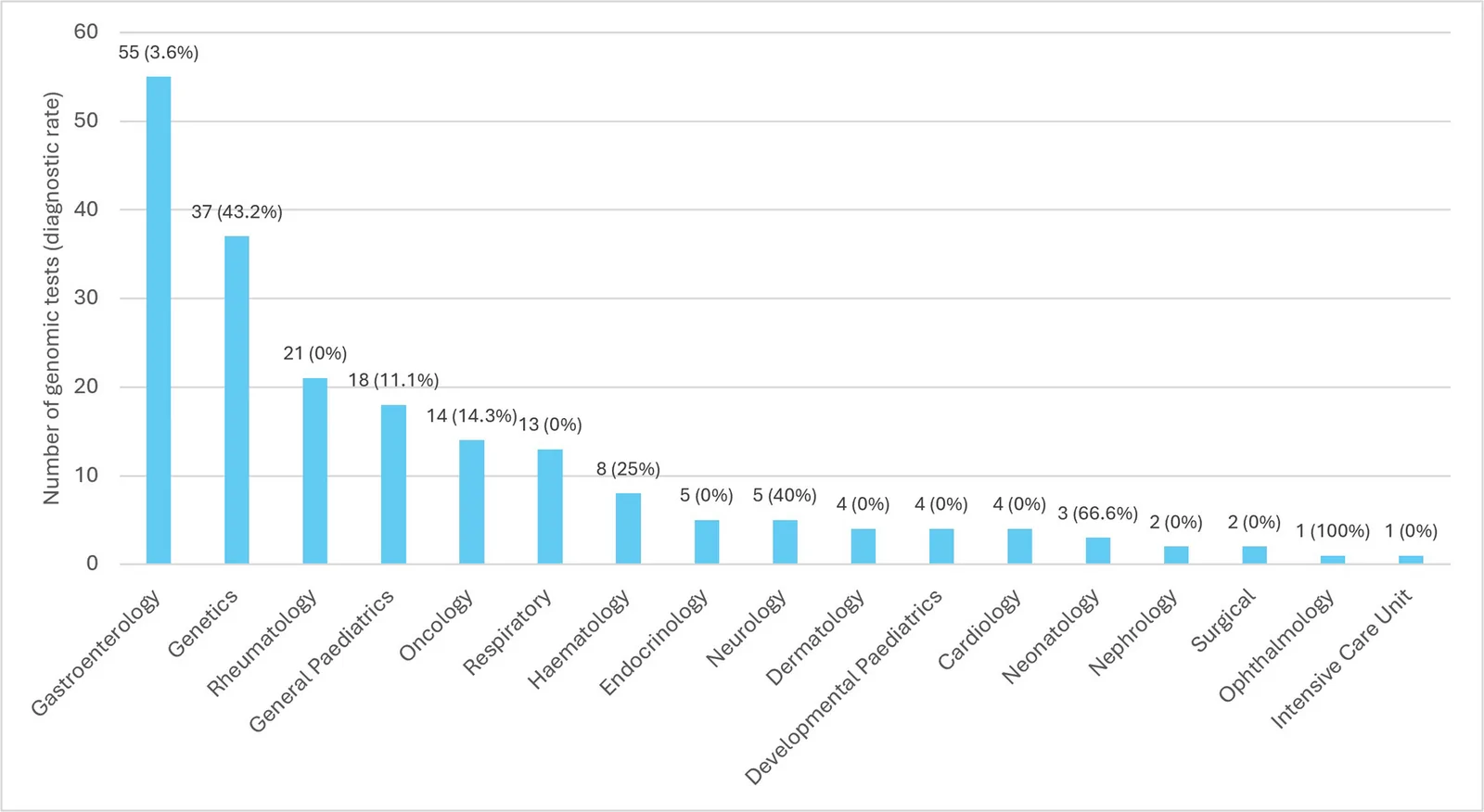
Genomic testing ordering departments. *Excludes testing initiated by an immunology clinician (see Fig. 5) and unknown cases. Includes duplicate testing

A total of 481 genomic tests were ordered for the 322 patients (mean:1.5 tests/patient, range:1–5 tests/patient), with 259 genomic tests performed pre-MoC (< 2021). More than one third of patients (*n* = 115;35.7%) received ≥ 2 genomic tests. Most genomic tests (*n* = 249;51.8%) were ordered by QPIAS immunologists, followed by gastroenterologists (*n* = 55;11.4%), geneticists (*n* = 37;7.7%), and rheumatologists (*n* = 21;4.4%) (Fig. 1).

Of the 115 individuals who received ≥ 2 genomic tests, 35 patients had duplicate tests, of which 16 were ordered by different specialties, 16 were ordered within the same specialty, and three where the ordering specialty was unknown. Examples of duplicate testing included, repeat familial testing for the same variant in the same individual (*n* = 1), and duplicate panel tests (*n* = 28), or single gene analysis (*n* = 6), all of which were conducted within a short timeframe (range:1 month to 2 years between tests). All duplicate test requests did not reference the original testing or provide a rationale to justify re-testing. Thus, excluding duplicate testing, there were 446 instances of unique genomic testing for the 322 patients, with 84 patients receiving ≥ 2 unique genomic tests (excluding research tests). Most of the unique genomic tests were arranged for diagnostic purposes (*n* = 425;95.3%), with the remainder pertaining to cascade testing of a pediatric relative, such as a sibling or cousin (*n* = 14;3.3%), segregation analysis to assist with VUS classification (*n* = 4;0.9%), or testing to inform HSCT donor eligibility (*n* = 3;0.7%).

Excluding duplicate tests, the most ordered genomic tests were panels and virtual panels (*n* = 294; 65.9%) (Table 2). Of the 125 virtual panels, testing was performed as a singleton (*n* = 73;58.4%), duo (*n* = 2;1.6%), or trio (*n* = 50;40.0%). Trio-testing was increasingly utilized for genomic tests (i.e., virtual panels, WES, and WGS) over time, with 66.7% (*n* = 52) of tests analyzed as a trio post-formal establishment of the MoC (≥ 2023), as compared to 18.1% prior (*n* = 13). Across the 294 panel and virtual panel tests, 352 gene panels were applied (*n* = 58 tests had ≥ 2 gene panels applied), with 288 (81.8%) of these gene panels specific to immunology (Table 2). The most frequently ordered immunology panels were immunological disorders super panel (*n* = 116;40.3%), IBD (*n* = 58;20.1%), and autoinflammatory disorders (*n* = 34;11.8%) **(**Table 2**)**. Non-immunology panels (*n* = 64) included symptoms involving other systems, including respiratory (*n* = 16), hematology (*n* = 15), metabolic (*n* = 5), gastroenterology (*n* = 5), neurology (*n* = 4), dermatology (*n* = 4), custom panel consisting of immunology and non-immunology genes (*n* = 4), pediatrics (*n* = 4), nephrology (*n* = 3), orthopedics (*n* = 3), or ophthalmology (*n* = 1).

Figure 3 shows the genomic testing trajectory for the 84 patients who had ≥ 2 unique tests, excluding research tests. Most commonly, patients received panel testing, including 43 in the first instance of testing (51.2%) and 42 in the second instance of testing (50.0%). All panel testing related to different gene lists. Few patients had expanded panels (*n* = 16), or re-analysis of a previous panel (*n* = 5). Ten patients underwent panel testing, CMA, or WES and were subsequently offered clinical WGS. Finally, one patient had two separate instances of familial testing, for two distinct familial variants.

**Table 2:** Unique instances of genomic testing

Genomic test modality (*n=*446^a^)		*n* (%)
	Panel	169 (37.9)
	Virtual panel	125 (28.0)
	Single gene	56 (12.6)
	Microarray	31 (7.0)
	Familial testing	21 (4.7)
	Whole exome sequencing	11 (2.5)
	Research testing	11 (2.5)
	Whole genome sequencing	13 (2.9)
	Other (e.g. mtDNA point mutation analysis, fragile X testing, somatic sequencing, karyotype)	7 (1.6)
	Unknown	2 (0.4)
Immunology panel type (*n*=288^b^)		*n* (%)
	Immunological disorders super panel	116 (40.3)
	Inflammatory bowel disease	58 (20.1)
	Autoinflammatory disorders	34 (11.8)
	Disorders of immune dysregulation	31 (10.8)
	Predominantly antibody deficiency	17 (5.9)
	Combined immunodeficiency	9 (3.1)
	Severe combined immune deficiency	7 (2.4)
	Phagocyte defects	4 (1.4)
	Complement deficiencies	4 (1.4)
	Defects of intrinsic and innate immunity	3 (1.0)
	Susceptibility to viral infections	3 (1.0)
	Chronic granulomatous disease	2 (0.7)

^a^Excludes duplicate testing

^b^Excludes non-immunology panels; counts total panels ordered, including cases with multiple panels per test

### Diagnostic Results

**Fig. 2:**
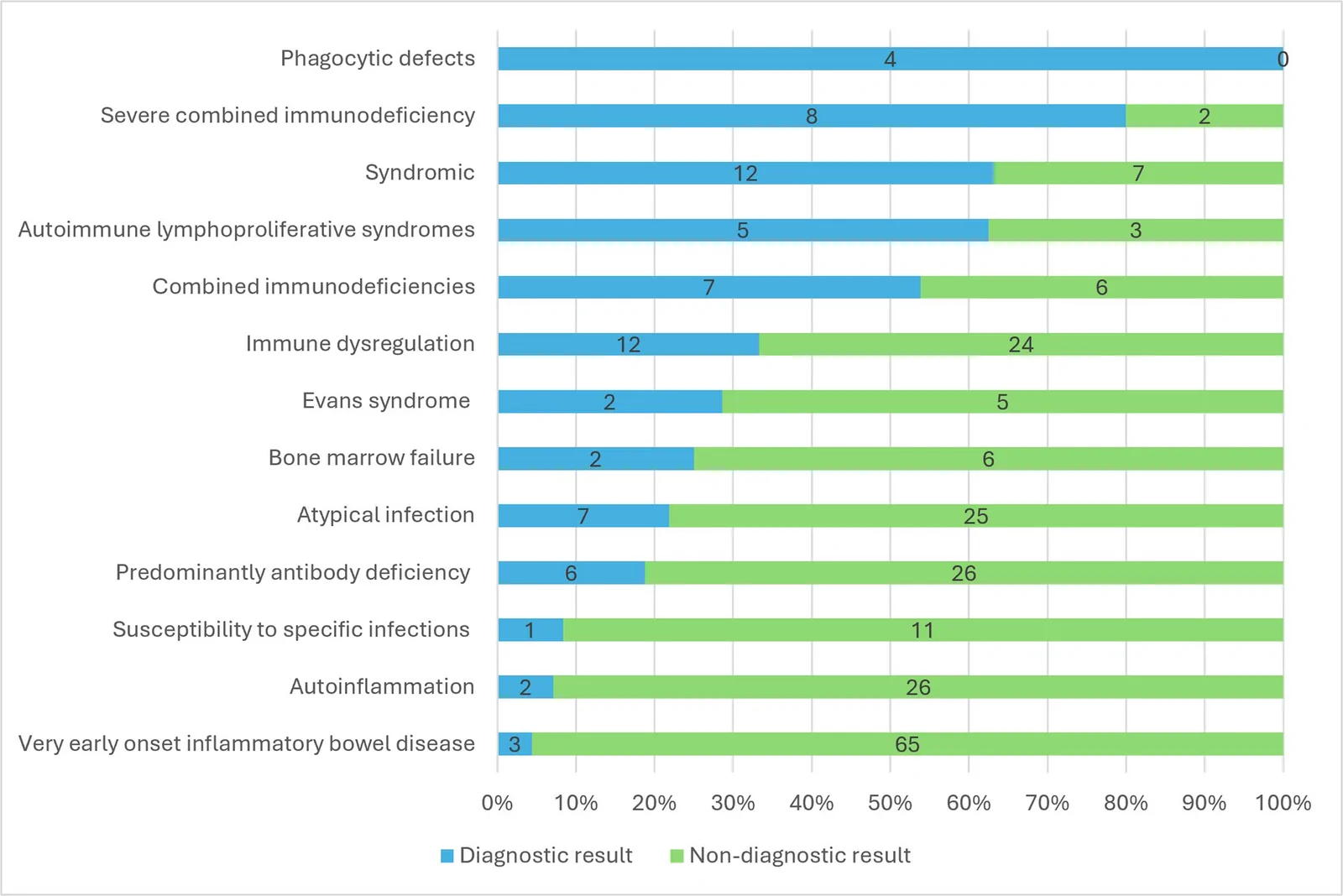
Clinical indications and diagnostic outcomes of genomic testing. *Excludes familial and unknown cases

A diagnostic result was identified for 86 patients (26.7%), of which 10 (3.1%) were syndromic cases. Excluding familial testing (*n* = 21), the diagnostic rate for probands was 20.2% (*n* = 65). Diagnostic yield varied according to the ordering department (Fig. 1). The diagnostic rate was highest among patients who were tested for phagocytic defects (*n* = 4/4;100%), SCID (*n* = 8/10;80%), and syndromic presentations (*n* = 12/19;63.2%) (Fig. 2). The lowest diagnostic yield was among patients who were tested for autoinflammation (*n* = 2/28;7.1%) and VEO-IBD (*n* = 3/68;4.4%). Excluding patients with VEO-IBD (*n* = 68) increased the diagnostic yield to 32.7% (*n* = 83).

**Fig. 3:**
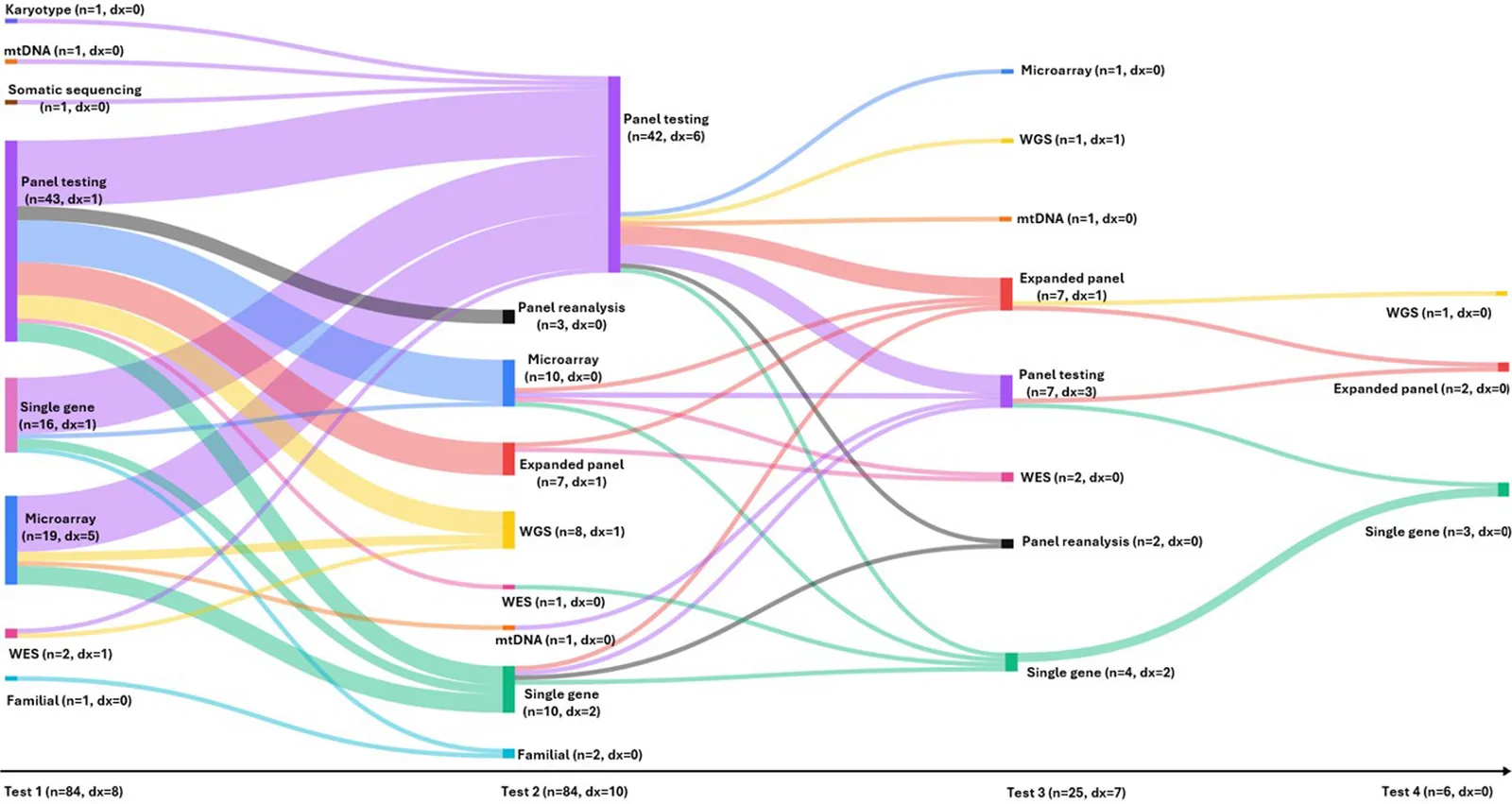
Distribution of patients across genomic testing pathways and diagnostic outcomes. *Excludes research tests. Panel testing inclusive of virtual panels. Dx: diagnosis; mtDNA: mitochondrial DNA point mutation analysis; WGS: whole genome sequencing; WES: whole exome sequencing

Of those that received a diagnostic result, most patients (*n* = 70; 81.4%) received a result in the first instance of genomic testing, and 16 (18.6%) in subsequent testing, which included alternate testing modality (*n* = 13), the application of a new panel (*n* = 1), expanded panel testing (*n* = 1), or a new single gene analyzed (*n* = 1) (Fig. 3). Sixteen patients who received a molecular diagnosis from initial genomic testing proceeded to undergo subsequent testing: eight to investigate clinical features not explained by the original genetic diagnosis, and eight appeared to have duplicate testing with no documented justification for re-testing.

**Fig. 4:**
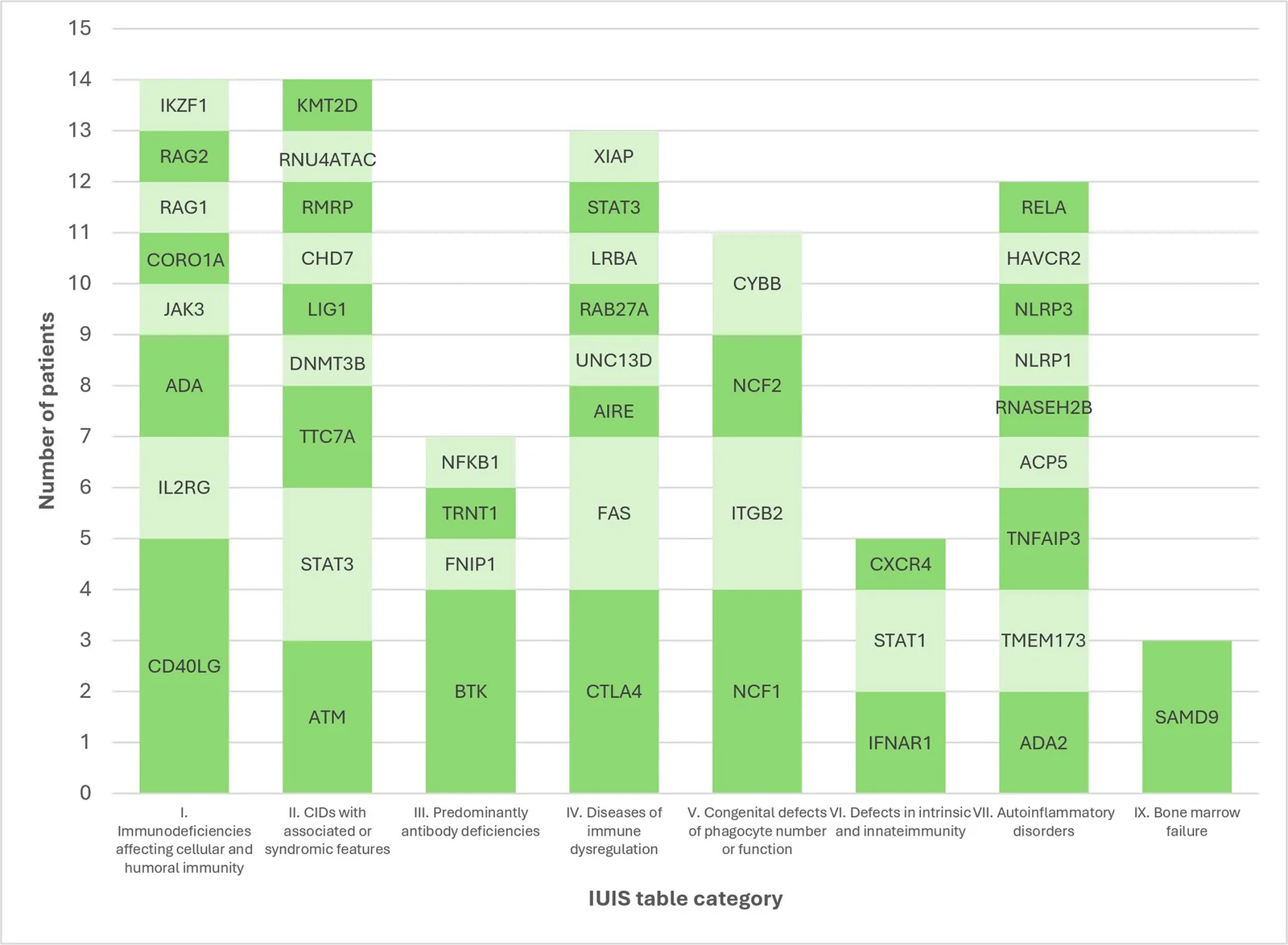
Implicated IUIS genes for diagnostic cases. CID: combined immunodeficiencies; IUIS: international union of immunological societies

Diagnostic results included variants in 45 IUIS genes across 79 patients (Supplementary Table 2). Notably, seven patients included in the overall diagnostic yield had a molecular diagnosis involving a non-IUIS gene (e.g., *UBE2A*, *WNT10A*) and were therefore not included in Supplementary Table 2. The most frequently implicated IUIS genes were *BTK* (*n* = 4), *CD40LG* (*n*= 5), *STAT3* (*n* = 4), *NCF1* (*n* = 4), and *CTLA4* (*n* = 4). ‘Combined immunodeficiencies (CID) with associated or syndromic features’ and ‘immunodeficiencies affecting cellular and humoral immunity’ were the two IUIS categories with the most patients (n = 14 patients respectively) (Fig. 4). Of molecular diagnoses included in the IUIS table (n = 79), 37 (46.8%) were autosomal recessive, 15 (19.0%) autosomal dominant, 14 (17.7%) X-linked, seven (8.9%) autosomal dominant gain-of-function (GOF), three (3.8%) autosomal dominant loss-of-function (LOF) with a dominant-negative effect, two (2.5%) autosomal dominant LOF, and one (1.3%) autosomal dominant with dominant-negative effect. Notably, *STAT3* variants were represented in two IUIS categories, reflecting both autosomal dominant GOF and LOF mechanisms.

Of the 446 unique instances of genomic testing, 101 tests (22.6%) yielded a VUS and 15 (3.4%) identified a risk allele, including those associated with increased risk for Crohn’s disease and common variable immunodeficiency (CVID) (e.g., *NOD2* and *TACI*). Risk alleles were not categorized as diagnostic, as such variants are not expected to cause the related presentation in isolation [27, 28]. Similarly, VUS were not categorized as diagnostic as it is recommended that such variants are not used to inform clinical decision making [29]. Four of the 36 patients referred to genomic research received a positive result: two enrolled in research as a first-line approach (pre-MoC; <2021), with subsequent clinical testing confirming pathogenic variants in *CXCR4* and *CTLA4*; and two patients who underwent functional studies to assist with the classification of VUS in *CTLA4*,[30] and *DOCK8 [*31].

Of the 10 (3.1%) deceased patients, six (60%) received a molecular diagnosis; implicated genes included *RAG1*,* CYBB*,* ADA2*,* G6PC3*,* NCF2*,* CHD7*, or *SAMD9*. Causes of death included complications following HSCT (*n* = 5) or sequelae of disease (*n* = 5).

### Patient Management Outcomes

Management change recommendations informed by molecular diagnosis were investigated for patients included in the IUIS table (*n* = 79) (Supplementary Table 2). A management change was recommended for 89.9% (*n* = 71) of patients. A major management change occurred for 69.6% of patients (*n* = 55), with ten patients receiving two forms of major management changes (*n* = 65 total major treatment changes). Changes included recommendation for HSCT (*n* = 29), other targeted treatment or management (*n* = 25), prophylaxis monitoring (*n* = 7), IRT (*n* = 3), and selection of conditioning regimen (*n* = 1). A minor management change occurred for 22.8% of patients (*n* = 18), all of which were changes to existing monitoring. Variations in management recommendations were observed among individuals with the same impacted gene and within familial cases, reflecting phenotypic variability and the timing of genomic testing. For instance, HSCT was initiated earlier in relatives due to a known familial variant, or a genomic diagnosis was established after targeted treatment had been delivered. No management change was recorded for 8.7% of patients (*n* = 7), as no change occurred or data could not be identified.

### Impact of Model of Care

All genomic tests that were excluded from the study due to results not being verifiable (*n* = 31) were ordered pre-formal establishment of the MoC (< 2023). Similarly, all 35 instances of duplicate testing occurred pre-MoC (< 2021) (*n* = 35/259;13.5%), compared to no duplicate testing post-MoC (≥ 2021) (*n* = 0/222;0%) (*p* < 0.05, chi-square test).

**Fig. 5:**
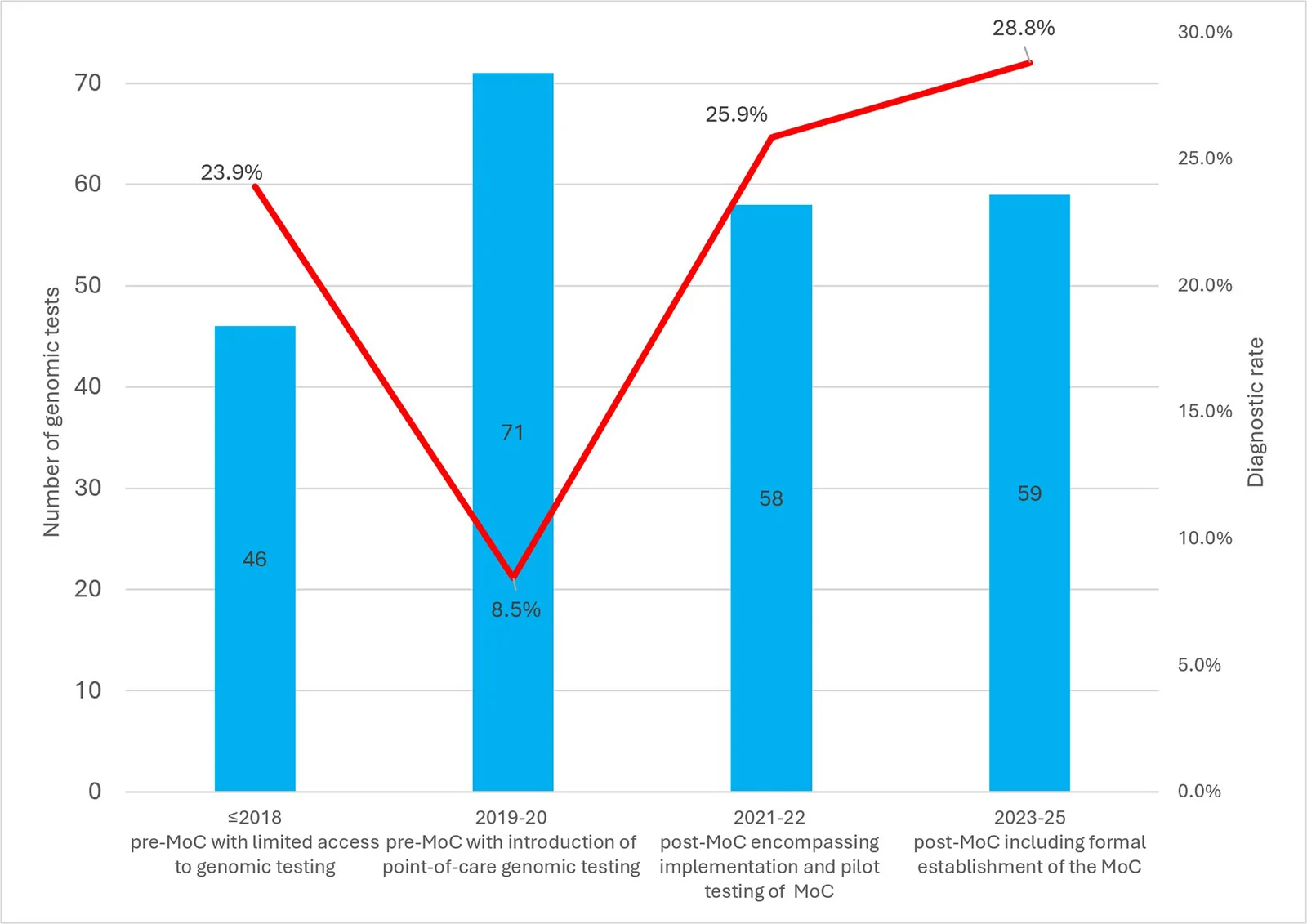
Diagnostic rate and genomic testing practices over time (*n* = 234). *Includes genomic testing initiated by QPIAS clinician. Excludes familial testing. MoC: model of care

Significant improvements in genomic testing practices (excluding familial testing) were noted for QPIAS initiated genomic testing (*n* = 234), including increased diagnostic yield over time (Fig. 5). Specifically, ≤ 2018 46 genomic tests were ordered with a diagnostic yield of 23.9% (*n* = 11). There was a substantial increase in genomic testing with the introduction of point-of-care genomic testing (2019–2020), which also correlated with a reduction in diagnostic yield (*n* = 6/71, 8.5%). Post-MoC (≥ 2021) the number of genomic tests ordered reduced, with the service delivering an average of 23.4 genomic tests per year between 2021 and 2025 and an average diagnostic rate of 27.4%. Overall, diagnostic yield significantly improved over time, increasing from 14.5% (*n* = 17/117) pre-MoC (< 2021) to 27.4% (*n* = 32/117) post-MoC (≥ 2021) (*p* < 0.05, chi-square test). Changes in testing laboratories for QPIAS initiated testing were also noted tests (excluding research and familial testing), with 16.6% (*n* = 18/108) of requested conducted by Pathology Queensland pre-MoC (< 2021), compared to 87.0% (*n* = 100/115) post-MoC (≥ 2021) (Supplementary Fig. 1).

Documentation of genomic consent increased over time (Supplementary Fig. 2). The rate of documented informed consent for QPIAS initiated testing significantly improved from 70.5% pre-MoC (< 2021) to 88.4% post-MoC (≥ 2021) (*p* < 0.05, chi-square test). Additionally, there were changes to rates of VUS reported in QPIAS initiated testing (inclusive of duplicate testing), pre-MoC implementation (< 2021) (*n* = 46/122;37.7%) as compared to post-MoC (≥ 2021) (*n* = 9/126;7.1%) (*p* < 0.05, chi-square test). All three patients who received a VUS in 2025 have been reviewed at the MDT and consented to research aimed at reclassifying these results.

## Discussion

This study characterizes an Australian pediatric IEI cohort and associated genomic testing practices, demonstrating the clinical and service effectiveness of implementing a multidisciplinary genomic MoC. Building on our previous feasibility assessment and parental evaluation of the MoC, [13, 32] findings demonstrate improvements in genomic testing practices associated with its implementation. Our diagnostic yield of 26.7% is comparable to other IEI studies (average yield: 29%, range 10–70%), [33] and most patients with an IUIS diagnostic result received a recommended management change (89.9%). Notably, the diagnostic rate varied over time, improving alongside the implementation of the MoC. Our findings build on previous work that provides guidance on facilitating IEI genomic testing, [8, 9] by offering recommendations for optimally integrating genomic testing within routine IEI pediatric healthcare (**Box 1**).

In keeping with international cohorts, [34–36] the diagnostic rate was highest among patients with phagocytic defects, SCID, and syndromic presentations. Conversely, the diagnostic rate was lowest among patients with VEO-IBD, a complex condition associated with IBD presenting under six years of age [37, 38]. Monogenic etiology of IBD is associated with a younger age-of-onset, and a more aggressive and treatment-refractory disease course. Our low diagnostic yield for VEO-IBD is likely reflective of initial broad eligibility criteria, that included offering genomic testing to all children diagnosed under six years of age, regardless of additional phenotypic features. Of the three patients with a confirmed molecular diagnosis for VEO-IBD in our cohort, all were diagnosed under two years of age and presented with severe symptoms (e.g., congenital diarrhea, suspected VEO-Behçet’s disease, and infantile enterocolitis). Revised eligibility criteria was implemented as part of the MoC in 2025 to automatically offer genomic testing to those with neonatal- and infantile-onset IBD (i.e., under two years of age), and review VEO-IBD diagnosed over two years of age for additional features of monogenic IBD and IEI, including immunophenotyping, family history, and disease severity [39]. This adaptive approach to genomic testing within our MoC is a key strength, whereby active monitoring of genomic data directly informs clinical practice and local service processes. More broadly, this approach has contributed to the development of IEI genomic testing recommendations [21].

This study highlights the diverse genomic testing modalities applied in pediatric IEI. Several factors influence the choice of test modality, including clinical presentation, suspected genomic mechanism, genomic technology availability, clinical and laboratory expertise, funding access, and patient consent [21]. While panel testing is well suited to IEI, given the ability to reliably phenotype patients, [40] WES and WGS are increasingly considered the most comprehensive and cost-effective approach to molecular diagnosis [41]. Specifically, employing a family-based trio approach for WES and WGS can further improve diagnostic yield and expedite the testing process [33]. Thus, where resources permit, adequate genetics expertise is available, and a family-based approach is feasible, trio-testing is considered the preferred first-line diagnostic test for pediatric IEI [33]. Additionally, our findings demonstrate the importance of ongoing investigations of non-diagnostic results, with 18.6% of diagnoses in our cohort occurring in subsequent testing, including research studies, expanded panels, and alternative testing modalities. Re-analysis of genomic data was limited to five patients within our cohort, which likely reflects the short timeframe of the MoC implementation, limiting opportunities for re-analysis at the recommended two to three year interval [42]. Given the rapid rate of gene discovery for IEI, re-analysis will play an important role in enhancing diagnostic yields and is considered an area for future improvement within this MoC [43, 44] Establishing collaborative partnerships with research entities is also important to resolving uncertain results and further investigating non-diagnostic results [45].

Our study adds to the evidence on the utility of genomic testing for IEI management, with 89.9% of patients with an IUIS molecular diagnosis receiving a recommended management change [9]. While genomic testing can alter IEI treatment, it is important that testing is delivered early in the disease course to mitigate irreversible disease related complications and avoid the use of contraindicated treatments. For example, HSCT requires conditioning treatment via chemotherapy to prepare for transplant. However, conditioning therapy should consider the molecular basis of disease to prevent toxicity (e.g. *LIG1* radiosensitive SCID) [46] Other therapeutic changes can include initiation of targeted treatment, such as commencing JAK inhibitors for *STAT1* and *STAT3* GoF variants, [47] anti-TNF inhibitors for *RelA*-related autoinflammatory disease, [48] PI3K-delta inhibitors for activated PI3K delta syndrome (APDS*)*, [49] and abatacept for treatment refractory CTLA4-haploinsufficiency [50]. Despite the impact of molecular diagnoses on IEI management, there is no Australian Medicare item number to fund genomic testing, which currently offers testing for 25 other conditions (e.g., pediatric intellectual disability, cardiovascular disease, metabolic conditions) [51]. Public testing costs for IEI are instead covered by individual hospitals and health services, potentially resulting in access disparities across geographical locations [52]. However, the availability of funding schemes alone does not guarantee uptake, as evidenced by the low use of existing Medicare rebates [53]. Therefore, it is important to develop implementation strategies to support the adoption of genomic testing and appropriate governance of testing, including an adequate workforce, genomic education, and MoC that incorporate genomic considerations.

While our study design did not permit association analysis to evaluate the impact of MoC on genomic testing practices, it is reasonable to infer certain patterns from the findings. The improved rate of documented patient consent is likely indicative of practice changes, including scheduled pre-test genetic counselling appointments and a new process for electronic filing of consent forms implemented by Queensland Health. The decreased rate of VUS is suggested to reflect changes in laboratory providers and strengthened collaboration with the local pathology team (i.e., Pathology Queensland), who attend the monthly MDT meetings, conduct variant prioritization meetings with clinicians, and have facilitated most IEI genomic testing post-MoC. Implementation of MDT case review also likely contributed to a reduction in duplicate testing and improved patient selection for genomic testing. Opportunities to further strengthen the MoC were also identified, including the need for greater cross-specialty collaboration, given that over half of genomic tests in this cohort were ordered by a non-immunology specialist [54]. These findings are consistent with previous studies, which reported an average of 7.9 specialists involved in IEI care [3]. Additional areas for improvement include ensuring documented informed consent across all cases, formalizing research testing pathways, and coordinating cascade testing for at-risk relatives.

**Box** **1**: Recommendations for delivering pediatric IEI genomic testing within routine healthcare

**Table:** 

1. Include service review processes to improve clinical guidelines, such as eligibility for genomic testing.
2. Where practical, utilize trio-WES or trio-WGS as a first-line test to improve diagnostic yield and optimize health service resources.
3. Establish processes for reviewal and re-analysis of negative or uncertain findings.
4. Increase collaboration with research entities and testing laboratories to resolve uncertain results or further investigate non-diagnostic results.
5. Consider the broader implications of diagnostic results, including the familial and reproductive considerations.
6. Ensure appropriate process for pre-test consent that supports patient decision-making and consent documentation.
7. Establish an MDT approach to incorporate cross-specialty collaboration and improve genomic testing practices.

Findings should be interpreted in line with the limitations of this study. The retrospective design required reliance on data primarily collected for clinical purposes, which is less accurate than data gathered prospectively [55]. Data on the genetic counselling and reproductive implications of molecular diagnoses could not be comprehensively collected, as related decisions frequently occurred within other clinical services and extended beyond the proband. Additionally, while the cohort was geographically and socio-economically representative of the wider Queensland population, significant inequities in access to genomic testing for pediatric IEI remain, [52] such as those related to ancestral background, which was not captured in this study. Nevertheless, a key strength of this study is its comprehensive audit approach, which enabled a detailed description of an IEI cohort and genomic testing utilization reflective of real-world clinical practice. While a formal economic evaluation was not undertaken, it is anticipated that these outcomes will be associated with cost savings for the public healthcare system. As genomic testing in pediatric IEI offers increasing clinical and personal utility, establishing evidence-based MoC becomes essential to supporting its optimal integration into routine healthcare and fully realizing these benefits. Our study demonstrates that integrating a multidisciplinary genomic MoC that considers the complexities of genomic testing can result in substantial improvements in genomic testing practices, patient care, and service delivery.

## Supplementary Information

Below is the link to the electronic supplementary material.

Supplementary Material 1

## Data Availability

De-identified data is available upon request to the corresponding author.
